# Anti-Inflammatory Effects of *Helianthus Tuberosus* L. Polysaccharide and Its Limited Gene Expression Profile

**DOI:** 10.3390/ijms26167885

**Published:** 2025-08-15

**Authors:** Evgenii Generalov, Leonid Yakovenko, Arkady Sinitsyn, Alexander Alekseev, Olga Sinitsyna, Khurshed Abduvosidov, Vladislav Minaichev, Liubov Generalova

**Affiliations:** 1 Faculty of Physics, Lomonosov Moscow State University, 119991 Moscow, Russia; yakovenko.lv@physics.msu.ru; 2 Faculty of Chemistry, Lomonosov Moscow State University, 119991 Moscow, Russia; apsinitsyn@gmail.com (A.S.); oasinitsyn@gmail.com (O.S.); 3 Federal Research Centre ‘Fundamental of Biotechnology’ of the Russian Academy of Sciences (FRC chBiotechnology RAS), 119071 Moscow, Russia; 4 Department of Human Morphology, Medical Institute of the Federal State Budgetary Educational Institution of Higher Education Russian Biotechnological University, 125080 Moscow, Russia; alekseevag@mgupp.ru (A.A.); sogdiana99@gmail.com (K.A.); 5 Institute of Theoretical and Experimental Biophysics, Russian Academy of Sciences, 142290 Pushchino, Russia; vminaychev@gmail.com; 6 Faculty of Medicine, Peoples’ Friendship University of Russia (RUDN University), 117198 Moscow, Russia; generals1100@mail.ru

**Keywords:** *Helianthus tuberosus* L. polysaccharide, carrageenan-induced oedema, pocket granuloma, transcriptome, anti-inflammatory activity

## Abstract

Previous studies have demonstrated that *Helianthus tuberosus* L. polysaccharide (HTLP) exhibits potent immunomodulating activity. The aim of this study was to investigate the molecular mechanisms underlying this activity and explore its potential applications in various anti-inflammatory models. We examined the anti-inflammatory potential of HTLP using in vitro and in vivo models. In vitro, we assessed the impact of HTLP on the expression of key inflammatory genes (*TNFA*, *IL1B*, *IL6*, *IL12B*, *IL23*, *CD40*, *CD80*, *CD274*, *CSF1*, and *NAMPT*) in lipopolysaccharide (LPS)-stimulated THP-1 cells. In vivo, we employed rat pocket granuloma and formalin- and carrageenan-induced oedema models. HTLP significantly reduced oedema volume in the in vivo models. In the carrageenan-induced oedema model, HTLP exhibited efficacy significantly higher than that of ibuprofen, reducing oedema by 76% at 8 h (*p* < 0.01). In the air pouch granuloma model, HTLP showed comparable anti-inflammatory activity to ibuprofen. In the formalin-induced oedema model, HTLP reduced oedema, demonstrating less efficacy than ibuprofen, with a reduction of 58% versus ibuprofen’s 65% (*p* < 0.001). The anti-inflammatory mechanism of HTLP involves not only the suppression of pro-inflammatory cytokine expression (*TNFA*, *IL1B*, *IL6*, *IL12B*, *IL23*, *CD40*, *CD80*, *CD274*, and *CSF1*) but also the activation of cell survival and cellular defence mechanisms (*NAMPT*) and the upregulation of the anti-inflammatory cytokine (*IL10)*. The observed biological activity of HTLP suggests its potential as a valuable therapeutic agent for inflammatory conditions. The combination of functional and molecular evidence demonstrates HTLP’s potent anti-inflammatory properties across multiple models, with efficacy approaching or exceeding that of ibuprofen in certain models. However, further studies are necessary to fully elucidate its mechanism of action and to evaluate its long-term efficacy and safety.

## 1. Introduction

The development of novel anti-inflammatory drugs is of considerable importance given the significant unmet clinical needs associated with a wide array of chronic diseases [1,2]. While modern anti-inflammatory therapies are often effective in managing symptoms for some patients, they frequently exhibit limited efficacy or adverse side effects or are ineffective for others [3,4,5]. Conditions such as rheumatoid arthritis, inflammatory bowel disease, lupus, and other chronic inflammatory disorders impose a significant global health burden. These conditions are characterised by chronic inflammation, which can cause tissue damage, functional impairment, and a lower quality of life [6,7]. The discovery of novel therapeutic agents with improved efficacy, targeted mechanisms of action, and lower toxicity profiles is critical for developing more effective and personalised treatment strategies. These novel drugs may also be useful in treating inflammatory diseases that do not respond to current treatments, as well as improving long-term management of chronic inflammatory conditions and, ultimately, contributing to a reduction in overall morbidity and mortality associated with these debilitating illnesses.

From this perspective, carbohydrates represent one promising group of biomolecules [8]. Polysaccharides, as representatives of carbohydrates, constitute a structurally diverse group of natural macromolecules renowned for their immunomodulatory properties [9]. These effects are mediated through their ability to interact with pattern-recognition receptors (PRRs) on immune cells, such as toll-like receptors (TLRs), C-type lectin receptors (CLRs), and complement receptors, triggering downstream signalling cascades, including NF-κB, MAPK, and PKC/PLC pathways [10,11].

Naturally occurring polysaccharides, sourced from plants, algae, and fungi, have exhibited substantial anti-inflammatory effects in both in vitro and in vivo models [9,12,13]. These effects are frequently mediated by the modulation of pro-inflammatory cytokine expression, such as TNF-α, IL-1β, and IL-6 [14,15,16]. Certain polysaccharides enhance the production and activity of anti-inflammatory cytokines, including IL-10 and TGF-β [17,18]. Polysaccharides, by modulating inflammatory cytokines, activate intracellular signalling cascades (MAPK, NF-κB, JAK-STAT, and PKC/PLC) known to play a key role in neuroinflammation, epilepsy, and pain [19,20,21,22].

Specific polysaccharide structures, such as sulphated polysaccharides and β-glucans, are associated with enhanced anti-inflammatory effects [23]. Beta-glucans, a class of natural polysaccharides, exert anti-inflammatory effects by engaging specific PRRs on immune cells [24]. The binding of beta-glucans to Dectin-1, a C-type lectin receptor expressed on macrophages and dendritic cells, triggers downstream signalling cascades that can modulate cytokine production [25]. Specifically, Dectin-1 activation by beta-glucans can promote the production of anti-inflammatory cytokines like IL-10 while dampening the release of pro-inflammatory mediators such as TNF-α [26].

Among sources of plant-derived polysaccharides, particular interest has been drawn to *Helianthus tuberosus* L. (another name is Jerusalem artichoke). Inulin-type fructans are its main components, demonstrating prebiotic effects by promoting the growth of beneficial gut bacteria and improving intestinal health [27]. Additionally, *Helianthus tuberosus* L. polysaccharides have shown antioxidant and immunomodulatory properties, potentially contributing to their health-promoting benefits [28,29]. Emerging research suggests that these polysaccharides may also possess anti-inflammatory and anti-tumour activities [30,31]. One of them, *Helianthus tuberosus* L. polysaccharide (HTLP)—a water-soluble β-glucan extracted from *Helianthus tuberosus* L.—has a molecular weight of 1–2 MDa, demonstrating biological activity in different models [32].

Previous studies have reported significant immunomodulating effects of HTLP. The data indicate a biphasic effect of polysaccharide concentration on TNF-α production in cell cultures. Low concentrations stimulated TNF-α production, whereas higher concentrations resulted in the suppression of TNF-α in a subset of donor cultures. The polysaccharide modulated IL-1 production by attenuating elevated levels and stimulating low-level expression [16]. Experimental studies have demonstrated that HTLP stimulates both innate and adaptive immune responses, including the activation of macrophages, NK cells, and cytotoxic T-cells. In murine models, HTLP has been shown to inhibit the metastasis of Lewis lung carcinoma by up to 80% and to enhance NK cell cytotoxic activity against tumour cells [33].

Moreover, HTLP displays radioprotective properties, safeguarding haematopoietic precursors and mitigating the detrimental effects of ionising radiation [34]. Its immunomodulatory effects are partially attributable to Dectin-1 and TLR-2/6 receptors, which are implicated in macrophage polarisation and the modulation of cytokine profiles, contingent upon cellular state and preactivation status [35]. This dual action not only facilitates the downregulation of pathological inflammation but also supports blood cellularity and the maintenance of normal immunological responses to pathogens [36].

While previous studies have demonstrated HTLP’s immunomodulatory effects, the precise molecular mechanisms by which it exerts its anti-inflammatory activity remain unclear. Furthermore, the therapeutic potential of HTLP in various in vivo models of inflammation has not been fully explored. The objective of this study is to elucidate the molecular mechanisms underpinning HTLP’s anti-inflammatory and immunomodulatory activity, with the further aim of exploring its potential therapeutic applications across diverse in vivo and in vitro models of inflammation.

## 2. Results

### 2.1. In Vitro Study of Anti-Inflammatory Activity

**Figure 1:**
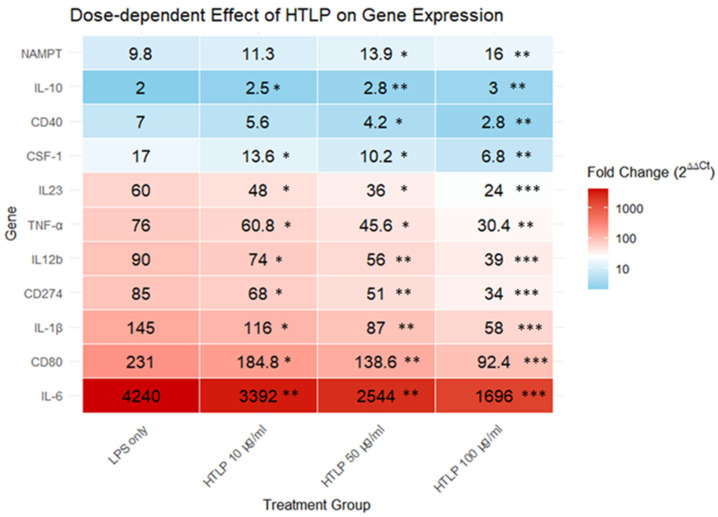
The dose-dependent effect of HTLP (10, 50, and 100 μg/mL) on the gene expression involved in the regulation of inflammatory processes and oxidative stress in LPS-induced THP-1 cells. mRNA expression levels were determined by quantitative real-time PCR. The *y*-axis represents the genes examined, while the *x*-axis represents the experimental groups (LPS alone and three doses of HTLP). The values within cells represent fold changes in gene expression (2^ΔΔCt^) compared to the control group. The Wilcoxon signed-rank test was used to compare each HTLP dose group to the LPS-only group. The number of biological replicates per group was *n* = 4. Asterisks represent statistically significant differences from the LPS-only group (* *p* < 0.05; ** *p* < 0.01; *** *p* < 0.001).

The exposure of differentiated THP-1-derived macrophages to lipopolysaccharide (LPS) induced a statistically significant upregulation in the expression of multiple genes. Quantitative PCR analysis revealed the following fold-changes in gene expression relative to unstimulated controls: *IL6*, 4240-fold; *CD80*, 231-fold; *IL1B*, 145-fold; *CD274*, 85-fold; *IL12B*, 90-fold; *TNFA*, 76-fold; *IL23*, 60-fold; *CSF1*, 17-fold; *CD40*, 7-fold; and *IL10*, 2-fold. Furthermore, LPS stimulation led to an increase in *NAMPT* gene expression from 5.1-fold to 9.8-fold, whereas combined LPS and HTLP treatment resulted in a further increase to 16-fold. Co-treatment with LPS and HTLP resulted in similar changes in gene expression, while *IL10* exhibited a 3-fold increase. The *NAMPT* gene encodes nicotinamide phosphoribosyltransferase, the rate-limiting enzyme in nicotinamide adenine dinucleotide biosynthesis. Notably, the secreted form of the NAMPT protein can function as a cytokine, modulating immune responses. The dose-dependent influence of HTLP (10, 50, and 100 μg/mL) on gene expression is demonstrated in Figure 1.

### 2.2. Carrageenan-Induced Oedema Model

**Figure 2:**
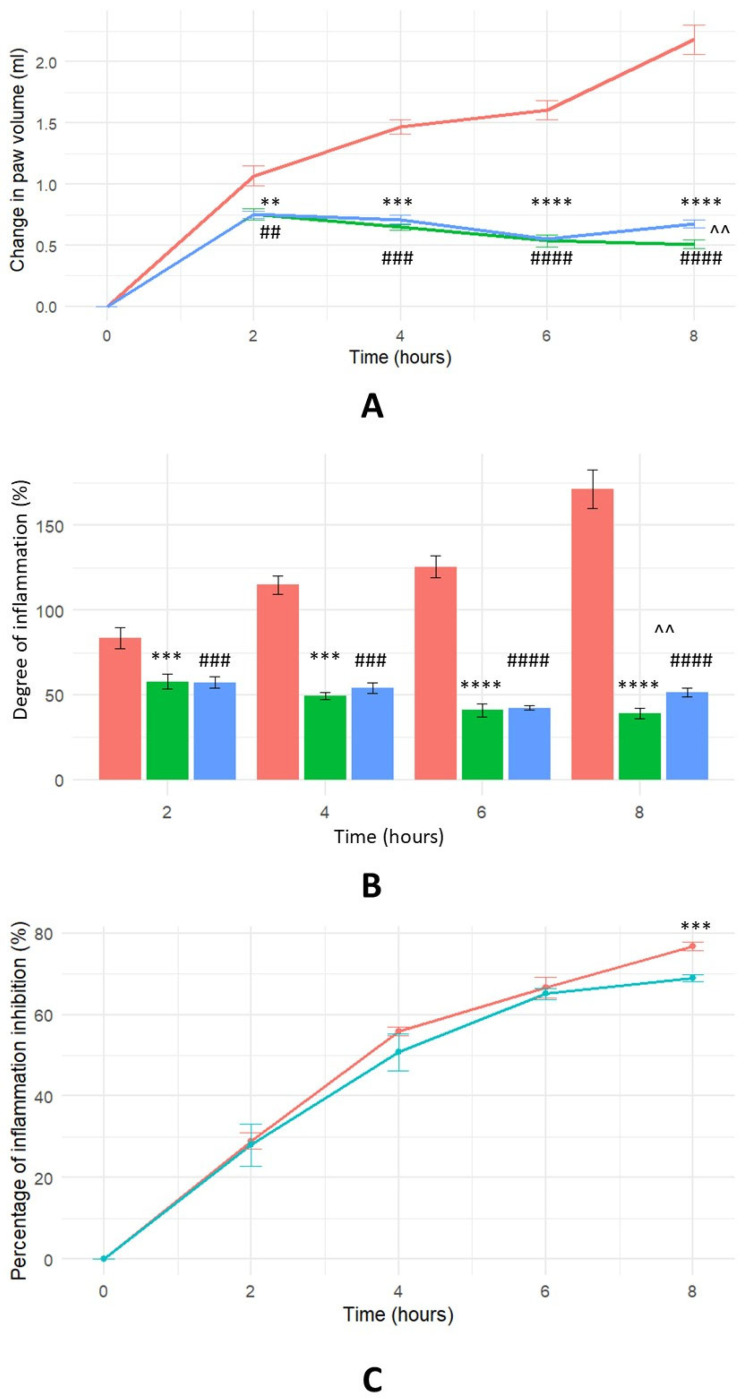
The time course of inflammation inhibition in a carrageenan-induced oedema model. For (**A**), the green colour represents 100 μg/rat HTLP administration; the blue colour represents 100 mg/kg ibuprofen administration; and the red colour represents saline control. For (**B**), the green colour represents 100 μg/rat HTLP administration; the blue colour represents 100 mg/kg ibuprofen administration; and the red colour represents saline control. For (**C**), the red colour represents 100 μg/rat HTLP administration and the blue colour represents 100 mg/kg ibuprofen administration. (**A**) Effect of HTLP and ibuprofen on paw volume in a carrageenan-induced oedema model. Each value represents the mean ± SEM of results from eight rats. Statistical significance was determined using one-way ANOVA followed by Tukey’s post hoc test. The significance level was adjusted for multiple comparisons. ** *p* < 0.01, *** *p* < 0.001, and **** *p* < 0.0001 compared to saline control; ^##^ *p* < 0.01, ^###^ *p* < 0.001, and ^####^ *p* < 0.0001 compared to saline control; ^^ *p* < 0.01 compared to the ibuprofen group. (**B**) Degree of inflammation in a carrageenan-induced oedema model. Each value represents the mean ± SEM of results from eight rats. Statistical significance was determined using one-way ANOVA followed by Tukey’s post hoc test. The significance level was adjusted for multiple comparisons. *** *p* < 0.001 and **** *p* < 0.0001 compared to saline control; ^###^ *p* < 0.001 and ^####^ *p* < 0.0001 compared to saline control; ^^ *p* < 0.01 HTLP compared to the ibuprofen group. (**C**) Percentage of inflammation inhibition by HTLP and ibuprofen over time in a carrageenan-induced oedema model. *** *p* < 0.001 HTLP compared to the ibuprofen group. The statistical significance level was set at *p* < 0.05 for all comparisons.

The anti-inflammatory potential of the polysaccharide HTLP was investigated using a rat model of carrageenan-induced paw oedema. Intraplantar injection of carrageenan elicited an acute inflammatory response, characterised by a statistically significant increase in paw volume relative to the control. Both substances (ibuprofen and HTLP) demonstrated anti-inflammatory properties. Paw volume measurements were serially acquired over an 8 h post-induction period to determine both the magnitude of oedema inhibition (Figure 2C) and the temporal kinetics of the anti-inflammatory effect (Figure 2B). Analysis of paw volume data (Figure 2A) revealed a statistically significant reduction in oedema following HTLP administration. The observed inhibition of paw oedema by HTLP exhibited efficacy significantly higher than that of ibuprofen. These findings demonstrate that HTLP effectively attenuates carrageenan-induced paw oedema by mitigating the increase in paw volume. These data support the potential utility of HTLP as a potential modulator of inflammatory processes, which can be potentially used as an anti-inflammatory drug.

Statistical analysis of the change in paw volume indicated significant differences for HTLP compared to the control group (at 6 and 8 h *p* < 0.0001), for ibuprofen compared to the control group (at 6 and 8 h *p* < 0.0001), and between HTLP and ibuprofen (at 8 h *p* < 0.001). These results suggest that both HTLP and ibuprofen possess anti-inflammatory activity. The maximum percentage of inflammation inhibition was observed for HTLP at 8 h (76%) and for ibuprofen (69%). A statistically significant difference was observed between the HTLP and ibuprofen treatment groups at 8 h (*p* < 0.001).

### 2.3. Air Pouch Granuloma Model

**Figure 3:**
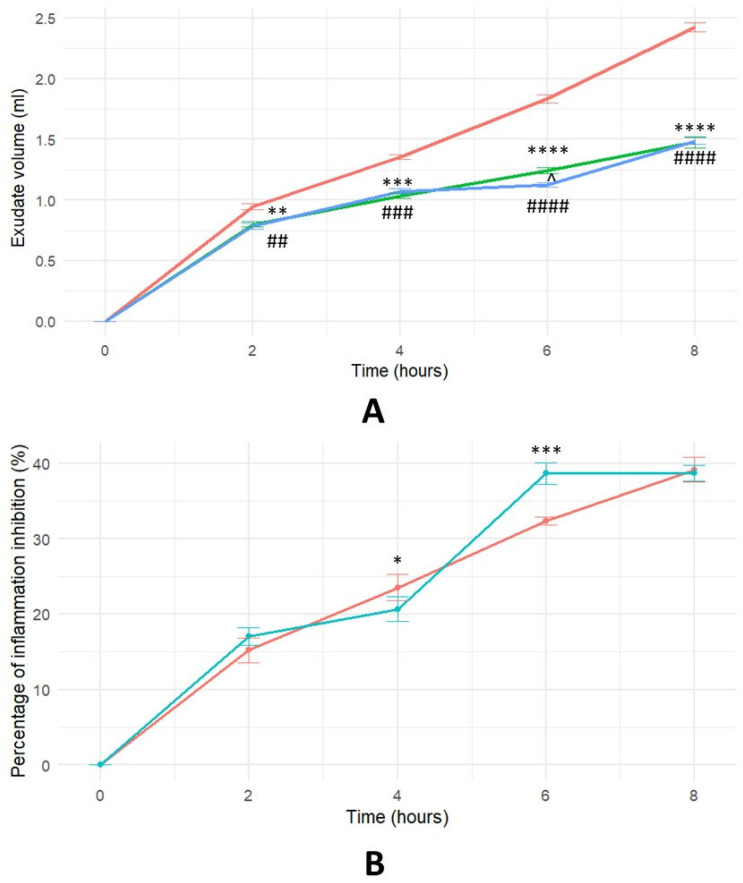
The time course of inflammation inhibition in a pocket granuloma model. For (**A**), the green colour represents 100 μg/rat HTLP administration; the blue colour represents 100 mg/kg ibuprofen administration; and the red colour represents saline control. For (**B**), the red colour represents 100 μg/rat HTLP administration and the blue colour represents 100 mg/kg ibuprofen administration. (**A**) Effect of HTLP and ibuprofen on exudate volume in an air pouch granuloma model. Each value represents the mean ± SEM of results from eight rats. Statistical significance was determined using one-way ANOVA followed by Tukey’s post hoc test. The significance level was adjusted for multiple comparisons. ** *p* < 0.01, *** *p* < 0.001, and **** *p* < 0.0001 compared to saline control; ^##^ *p* < 0.01, ^###^ *p* < 0.001, and ^####^ *p* < 0.0001 compared to saline control; ^ *p* < 0.01 HTLP compared to the ibuprofen group. (**B**) Percentage of inflammation inhibition by HTLP and ibuprofen over time in an air pouch granuloma model. * *p* < 0.05 and *** *p* < 0.001 HTLP compared to the ibuprofen group. The statistical significance level was set at *p* < 0.05 for all comparisons.

The anti-inflammatory effects of HTLP were further assessed using a rat pocket granuloma model. The dynamics of inflammation suppression (Figure 3) were evaluated by comparing the effects of HTLP to those of a positive control (ibuprofen) and a negative control (saline solution). Figure 3A shows a decrease in exudate volume after treatment with ibuprofen (*p* < 0.01 at 2 h, *p* < 0.001 at 4 h, *p* < 0.0001 at 6 and 8 h) and HTLP (*p* < 0.01 at 2 h, *p* < 0.001 at 4 h, *p* < 0.0001 at 6 and 8 h) compared to the saline control. No statistically significant differences were observed between the HTLP and ibuprofen treatment groups at 2, 4, and 8 h (*p* > 0.05). At the same time, a statistically significant difference was observed between the HTLP and ibuprofen treatment groups at 6 h (*p* < 0.05). These findings indicate that both the HTLP and ibuprofen treatments resulted in a substantial reduction in oedema volume, in contrast to the control group, which exhibited sustained inflammatory activity.

Figure 3B illustrates the time course of the percentage of inflammation inhibition. These findings indicate that both HTLP and ibuprofen treatments resulted in a moderate reduction in oedema volume. The percentage of inflammation inhibition for HTLP was maximal at 8 h (38%); for ibuprofen, it reached a plateau after 6 h (39%). A statistically significant difference was observed between the HTLP and ibuprofen treatment groups at 4 and 6 h (*p* < 0.05, *p* < 0.001, respectively).

These data demonstrate that HTLP possesses specific anti-inflammatory activity, with effects approaching those of ibuprofen and significantly exceeding that observed in the control group.

### 2.4. Formalin-Induced Oedema

**Figure 4:**
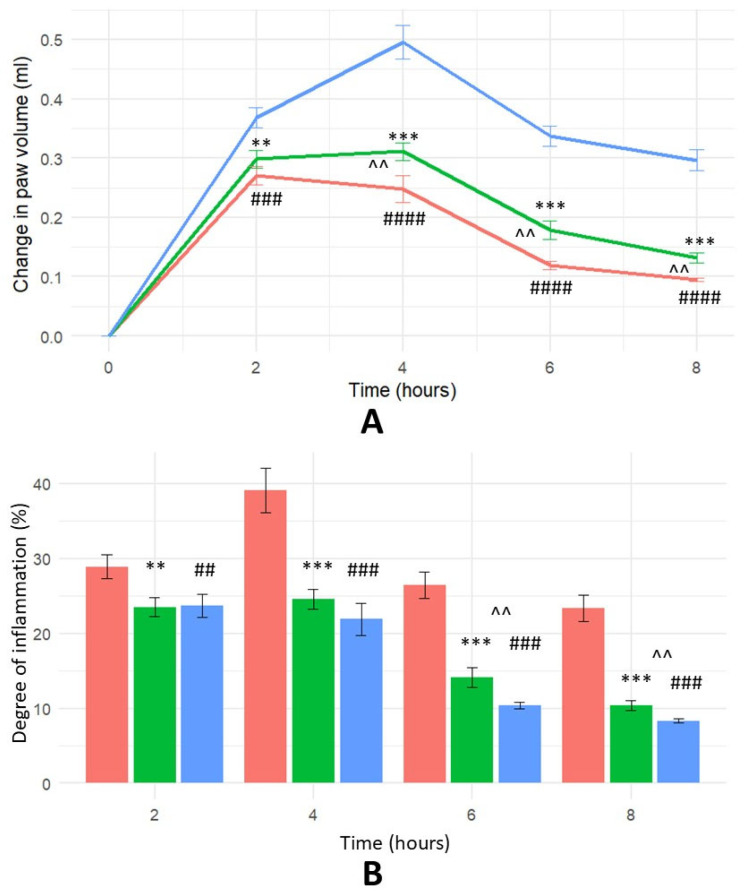

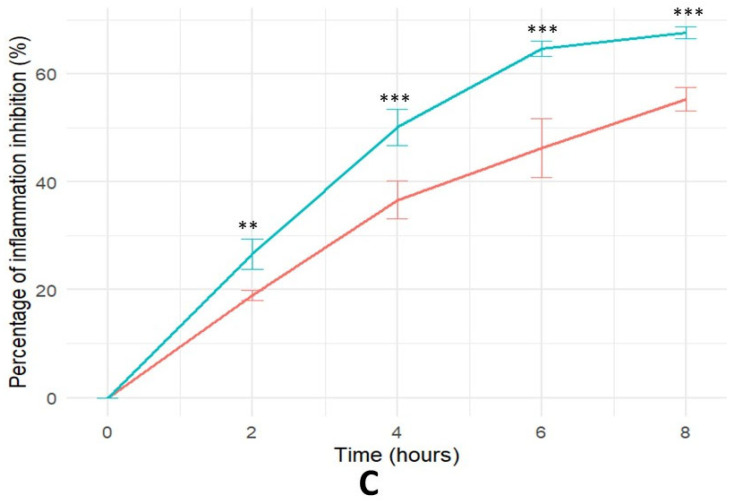
The time course of inflammation inhibition in a formalin-induced oedema model. For (**A**), the green colour represents 100 μg/rat HTLP administration; the red colour represents 100 mg/kg ibuprofen administration; and the blue colour represents saline control. For (**B**), the green colour represents 100 μg/rat HTLP administration; the blue colour represents 100 mg/kg ibuprofen administration; and the red colour represents saline control. For (**C**), the red colour represents 100 μg/rat HTLP administration and the blue colour represents 100 mg/kg ibuprofen administration. (**A**) Effect of HTLP and ibuprofen on paw volume in a formalin-induced oedema model. Each value represents the mean ± SEM of results from eight rats. Statistical significance was determined using one-way ANOVA followed by Tukey’s post hoc test. The significance level was adjusted for multiple comparisons. ** *p* < 0.01 and *** *p* < 0.001 compared to saline control; ^###^ *p* < 0.001 and ^####^ *p* < 0.0001 compared to saline control; ^^ *p* < 0.01 compared to the ibuprofen group. (**B**) Degree of inflammation in a formalin-induced oedema model. Each value represents the mean ± SEM of results from eight rats. Statistical significance was determined using one-way ANOVA followed by Tukey’s post hoc test. The significance level was adjusted for multiple comparisons. ** *p* < 0.01 and *** *p* < 0.001 compared to saline control; ^##^ *p* < 0.01 and ^###^ *p* < 0.001 compared to saline control; ^^ *p* < 0.01 compared to the ibuprofen group. (**C**) Percentage of inflammation inhibition by HTLP and ibuprofen over time in a formalin-induced oedema model. ** *p* < 0.01 and *** *p* < 0.001 compared to the ibuprofen group. The statistical significance level was set at *p* < 0.05 for all comparisons.

In rats, intraplantar injection of formalin induces acute paw oedema. Figure 4 shows the effects of HTLP on formalin-induced oedema. Among the tested solutions, the highest anti-inflammatory activity was shown by ibuprofen, as measured by changes in paw volume and the calculated percentage of inflammation inhibition, which was calculated using a formula similar to Equations (1) and (2). Figure 4 illustrates formalin-induced inflammation in the rat hind paw. Both HTLP and ibuprofen possess anti-inflammatory activity.

This study revealed statistically significant differences in the anti-inflammatory efficacy of the tested substances. HTLP exhibited a marked and sustained anti-inflammatory effect, displaying an approximately linear increase in activity throughout the observation period. Statistical analysis revealed significant differences (Figure 4A) in the oedema volume between the HTLP and control groups (at 2 h *p* < 0.01, at 4, 6 and 8 h *p* < 0.001), the ibuprofen and control groups (at 2 h *p* < 0.001, at 4, 6 and 8 h *p* < 0.0001), and the HTLP and ibuprofen groups at 4, 6, and 8 h (*p* < 0.01).

The minimal percentage increase in the oedema volume was observed after 8 h when compared to the control group (Figure 4B). Statistical analysis revealed significant differences in the oedema volume between the HTLP and control groups (at 2 h *p* < 0.01, at 4, 6 and 8 h *p* < 0.001), the ibuprofen and control groups (at 2 h *p* < 0.001, at 4, 6 and 8 h *p* < 0.0001), and the HTLP and ibuprofen groups at 6 and 8 h (*p* < 0.01).

However, analysis of the percentage of inflammation inhibition (Figure 4C) indicated that ibuprofen’s efficacy was greater than that of HTLP at 2 (*p* < 0.01), 4, 6, and 8 h (*p* < 0.001). At the 8 h endpoint, the percentage of inflammation inhibition for HTLP reached 58%, whereas ibuprofen demonstrated a percentage of inflammation inhibition of approximately 65%. These results indicate that, although both substances exhibited marked and sustained anti-inflammatory activity, ibuprofen was more effective than HTLP in this model.

## 3. Discussion

HTLP administration led to a significant decrease in oedema volume. This suggests that the compound possesses an anti-exudative effect. In the air pouch granuloma model, inflammation decreased at a similar rate in the HTLP and ibuprofen groups. At the same time, in the carrageenan-induced oedema model, HTLP showed significantly higher anti-inflammatory activity than ibuprofen. Simultaneously, both treatment groups were significantly more effective than the control group, which only received saline. In the formalin-induced oedema model, ibuprofen showed stronger anti-inflammatory activity than HTLP.

The expression of key inflammatory genes, to assess the underlying molecular mechanisms, was analysed by qPCR. The analysis revealed a significant decrease in the levels of pro-inflammatory cytokine gene expression (*TNFA*, *IL1B*, *IL6*, *IL12B*, *IL23*, *CD40*, *CD80*) and a concurrent increase in the expression of the anti-inflammatory cytokine genes *IL10* and *CD274* in cells treated with HTLP, as compared to LPS-activated cells.

The *TNFA* gene encodes a key pro-inflammatory cytokine produced primarily by macrophages and T-cells. TNF-α plays a central role in systemic inflammation, increases vascular permeability, promotes leukocyte recruitment, and stimulates the production of other cytokines and chemokines [37]. *IL1B* is a gene of a well-known pro-inflammatory cytokine primarily produced by activated macrophages, monocytes, and dendritic cells. IL-1b’s primary role in inflammation is to initiate and amplify the inflammatory response, increase vascular permeability, recruit immune cells to the site of inflammation, and stimulate the production of other pro-inflammatory cytokines. *IL6* is a gene of the IL-6 cytokine, which is also a pro-inflammatory cytokine with pleiotropic biological activity, produced by a wide range of cells, including immune cells, fibroblasts, and endothelial cells. IL-6 contributes to inflammation by stimulating the production of acute-phase proteins, activating immune cells, influencing B- and T-cell differentiation, and potentially promoting both Th17 and regulatory T-cell (Treg) responses, depending on the context [38]. IL-12 (encoded by the *IL12* gene) is a pro-inflammatory cytokine produced by antigen-presenting cells (APCs) like dendritic cells and macrophages and plays a crucial role in bridging innate and adaptive immunity, stimulating the production of IFN-γ by T- and NK-cells [39]. The *IL23* gene encodes the IL-23 pro-inflammatory cytokine primarily produced by APCs; promotes the survival, proliferation, and activation of Th17 cells; and promotes the production of other pro-inflammatory cytokines, such as TNF-α and IL-6 [40]. The *CD40* gene expresses the CD40 protein, which is a co-stimulatory protein expressed in APCs, such as B-cells, macrophages, and dendritic cells. It interacts with its CD40L ligand, which is expressed in activated T-cells. This interaction is crucial for T-cell-dependent B-cell activation, immunoglobulin class switching, and antibody production. CD40 signalling also enhances the antigen-presenting capacity of APCs, leading to increased T-cell activation [41]. The CD80 gene encodes the CD80 protein, which is a co-stimulatory molecule expressed in APCs. It interacts with CD28 in T-cells to provide a crucial co-stimulatory signal required for T-cell activation and proliferation. Without CD80 co-stimulation, T-cells may become anergic or undergo apoptosis [42]. These findings therefore support the in vivo observations of reduced inflammation following HTLP treatment.

At the same time, treatment with HTLP stimulated the expression of the anti-inflammatory cytokine gene *IL10*. The *IL10* gene encodes a key anti-inflammatory IL-10 protein produced by various immune cells, including regulatory T-cells (Tregs), macrophages, and B-cells. It inhibits the production of pro-inflammatory cytokines (such as TNF-α, IL-1β, and IL-6) by macrophages and dendritic cells, suppresses T-cell proliferation and activation, and promotes the differentiation of Tregs [43].

CSF-1 (M-CSF): CSF-1 is a haematopoietic growth factor that primarily stimulates the proliferation, differentiation, and survival of monocytes, macrophages, and osteoclasts. It is essential for the development and maintenance of the monocyte–macrophage lineage. CSF-1 can have both pro- and anti-inflammatory effects, depending on the context. In some situations, it can promote inflammation by recruiting macrophages to the site of inflammation and enhancing their phagocytic activity. CSF-1 is involved in bone remodelling, immune regulation, and wound healing [44].

The *NAMPT* gene encodes nicotinamide phosphoribosyltransferase (NAMPT), a key enzyme catalysing the rate-limiting step in the biosynthesis of nicotinamide adenine dinucleotide (NAD+), a crucial coenzyme for cellular metabolism, signalling, and genome stability. NAMPT is secreted by various cell types, including immune cells, adipocytes, and hepatocytes, functioning both intracellularly and as an extracellular cytokine (visfatin). Intracellular NAMPT promotes cell survival and defence against various cellular stressors, such as oxidative stress and nutrient deprivation, by activating sirtuins and regulating mitochondrial function [45].

These molecular data correlate with the functional outcomes, confirming that HTLP effectively modulates the inflammatory cascade by suppressing the production of pro-inflammatory mediators and activating protective mechanisms.

The observed anti-inflammatory kinetics of HTLP warrant further investigation. The absence of a perceptible plateau in oedema reduction by the 8th hour of observation suggests the potential for a sustained duration of action beyond the measured timeframe.

Given the diverse etiological mechanisms underlying air pouch granuloma, carrageenan-induced oedema, and formalin-induced oedema, HTLP demonstrates a broad-spectrum anti-inflammatory profile. The efficacy of HTLP in reducing formalin-induced oedema, carrageenan-induced oedema, and air pouch granuloma formation implies that HTLP’s mechanism of action targets multiple pathways involved in inflammatory processes. This multimodal activity suggests HTLP’s potential to modulate inflammatory cascades irrespective of the initiating stimulus.

Previous studies [16,32,33,34,35,36] have shown that HTLP is a β-glycan exhibiting polyelectrolyte properties, with primarily β-(1→4)- and β-(1→3)-glycosidic bonds and a high molecular weight of 1–2 MDa. These structural features are consistent with its observed ability to interact with Dectin-1 and TLR-2/6 receptors, which are likely key mediators of its biological activities. However, further investigations must be conducted for more in-depth structural analyses to fully characterise HTLP and further investigate the relationship between its specific structural features and anti-inflammatory activity.

Despite its positive results, this study has several limitations. Firstly, this study investigates only one dose of HTLP in vivo without assessing the dose-dependent behaviour of the substances. Secondly, the gene expression analysis was conducted primarily at the mRNA level using qPCR without validation at the protein level, leaving questions regarding the functional implementation of the observed changes. Additionally, the observation period was limited to 8 h, which does not allow for the assessment of the long-term effects and safety of HTLP, and the molecular mechanisms underlying its action require further investigation using more comprehensive approaches.

The data obtained demonstrate that the polysaccharide HTLP exerts a potent anti-inflammatory effect, not only by suppressing pro-inflammatory signals but also by activating anti-inflammatory genes and cellular defence mechanisms. The broad action of HTLP, confirmed by in vitro results in the LPS-induced inflammation model and further supported by in vivo experiments using formalin- and carrageenan-induced oedema and pocket granuloma models in rats, highlights its potential as a promising therapeutic agent for the treatment of inflammatory diseases.

## 4. Materials and Methods

### 4.1. Studied Substance

This study examined the specific activity of the polysaccharide complex HTLP with a molecular weight of 1–2 MDa, consisting predominantly of uronic acids, glucose, and galactose, as well as other monosaccharides, including arabinose, rhamnose, mannose, and xylose. HTLP was isolated and purified from *Helianthus tuberosus* L. using cross-filtration, chromatography, and membrane purification methods according to the methodology described previously [36]. The selection of HTLP dosages for the studies (10, 50, and 100 µg/mL for in vitro and 100 µg/rat for in vivo) was based on preliminary experiments evaluating the toxicity and efficacy of the substance, as well as literature data on similar polysaccharide complexes [16,32,36]. In vitro studies demonstrated that 100 µg/mL of HTLP was the most effective concentration for modulating cytokine expression. Specifically, in peripheral blood monocyte (PBM) cells, HTLP at 100 µg/mL modulated the levels of pro-inflammatory cytokines IL-1, IL-6, and TNF-α (measured by ELISA). Furthermore, HTLP also stimulated interferon expression in both in vitro and in vivo models. In vitro, doses of 10–50 µg/mL stimulated interferon production in fibroblasts (M-19). In vivo, we found that similar doses resulted in an increase in interferon levels in rat serum (C57Black/6) after administration. Moreover, 100 µg/rat showed no evidence of significant toxicity (in acute toxicity, the mass and cellularity of the immune system and cell viability of the lymphoid organs) in preliminary in vivo assessments. In preliminary studies, doses up to 1000 µg/rat showed no significant changes in allergic reaction, liver enzymes, or other markers of toxicity.

### 4.2. In Vivo Study of Anti-Inflammatory Activity

#### 4.2.1. Experimental Animals

This study used male Wistar rats weighing 200–240 g (purchased from the branch of the Institute of Bioorganic Chemistry named after M.M. Shemyakin and Yu.A. Ovchinnikov RAS, Moscow, Russia), with 8 animals per group. The animals were housed in individually ventilated cages at a temperature of 22 ± 2 °C, relative humidity of 55 ± 10%, and a 12 h light/dark cycle, in accordance with the requirements of Directive 2010/63/EU of the European Parliament and of the Council of 22 September 2010, regulating the protection of animals used for scientific purposes [46].

All experimental protocols strictly adhered to the ethical principles outlined in the Declaration of Helsinki (1964) and its subsequent amendments. Animal studies were conducted in accordance with the guidelines of the Federation of European Laboratory Animal Science Associations (FELASA) [47] and the European Convention for the Protection of Vertebrate Animals (Strasbourg, 18 March 1986). The animal study protocol was approved by the Ethics Committee of the Institute of Theoretical and Experimental Biophysics of the Russian Academy of Sciences (protocol #36/2025 of 3 March 2025).

#### 4.2.2. Carrageenan-Induced Oedema Model

To assess the anti-inflammatory activity of HTLP, the carrageenan-induced oedema model [48], widely used for the primary screening of potential anti-inflammatory agents, was employed. The animals received a subplantar injection of 0.1 mL of 1% carrageenan gel into the right hind paw. The left hind paw, which did not receive the carrageenan injection, served as a control. HTLP solutions were administered intraperitoneally 1 h prior to the carrageenan injection.

Oedema intensity was measured at 2, 4, 6, and 8 h after induction (time points chosen based on the dynamics of carrageenan-induced oedema). The paw volume was measured using a digital plethysmometer (Ugo Basile, Gemonio, VA, Italy) by immersing the paw up to the tarsal region in a water-filled measurement chamber. Each measurement was taken three times at 1 min intervals, and the average value was calculated. The percentage of inflammation inhibition (*PI*) was calculated using the following formula [49]:

(1) $$PI=(1-\frac{V_{o}}{V_{k}})\;*\;100$$

where *V_o_* represents the increase in oedema volume in the treated group, and *V_k_* represents the increase in oedema volume in the control group.

The degree of inflammation (*DI*) induced was evaluated according to the following formula [49]:

(2) $$DI=(\frac{P_{o}-P_{k}}{P_{k}})\;*\;100$$

where *P_o_* represents the volume of the right hind paw after inflammation induction, and *P_k_* represents the volume of the right hind paw before inflammation induction.

The following groups were formed:

1. Control group—animals received a sterile 0.9% NaCl solution intraperitoneally 1 h prior to the carrageenan injection (*n* = 8);
2. Experimental group—animals received a sterile 0.9% NaCl solution containing HTLP at a dose of 100 µg/rat intraperitoneally 1 h before carrageenan administration (*n* = 8);
3. Comparison group—animals received ibuprofen (a well-known anti-inflammatory agent) at a dose of 100 mg/kg intraperitoneally 1 h before carrageenan administration (*n* = 8).

#### 4.2.3. Air Pouch Granuloma Model

The air pouch granuloma model was also used to comprehensively assess HTLP’s anti-inflammatory activity at different stages of the inflammatory process [50]. The animals were given 20 cm^3^ of sterile air subcutaneously in the interscapular region to form an air pouch. Then, 0.5 mL of a 50% oil solution of turpentine was injected into the pouch to induce inflammation. Sterile HTLP solutions were administered intraperitoneally one hour before the turpentine injection.

Oedema was measured two, four, six, and eight hours after inflammation induction. The volume of exudate in the granuloma was determined by puncturing and aspirating fluid from the air pouch with a sterile syringe and needle, then measuring volume with a graduated tube.

The following groups were formed:

1. Control group—animals received a sterile 0.9% NaCl solution intraperitoneally 1 h prior to the turpentine injection (*n* = 8);
2. Experimental group—animals received a sterile 0.9% NaCl solution containing HTLP at a dose of 100 µg/rat intraperitoneally 1 h prior to the turpentine injection (*n* = 8);
3. Comparison group—animals received ibuprofen at a dose of 100 mg/kg intraperitoneally 1 h prior to the turpentine injection (*n* = 8).

#### 4.2.4. Formalin-Induced Oedema Model

Animals were injected subplantarly with 0.1 mL of 2% aqueous formalin solution [51]. To assess inflammation, the volume of oedema at the injection site was assessed before and after injection using a digital plethysmometer. The percentage of inflammation inhibition and the degree of inflammation were calculated using Formulae (1) and (2) provided above.

The following groups were formed:

1. Control group—animals received a sterile 0.9% NaCl solution intraperitoneally 1 h before formalin administration (*n* = 8);
2. Experimental group—animals received a sterile 0.9% NaCl solution containing HTLP at a dose of 100 µg/rat intraperitoneally 1 h before formalin administration (*n* = 8);
3. Comparison group—animals received ibuprofen at a dose of 100 mg/kg intraperitoneally 1 h before formalin administration (*n* = 8).

HTLP solutions (1 mL) were administered intraperitoneally 1 h before formalin administration. The percentage of inflammation inhibition was calculated 2, 4, 6, and 8 h after oedema induction using Formulae (1) and (2).

### 4.3. In Vitro Study of Anti-Inflammatory Activity

#### 4.3.1. Cell Line and Culture Conditions

This study used the THP-1 cell line (human monocytes) obtained from the American Type Culture Collection (ATCC, catalogue number TIB-202). The cells were cultured in RPMI-1640 medium supplemented with 10% foetal bovine serum (FBS), 2 mM L-glutamine, 100 U/mL penicillin, and 100 µg/mL streptomycin (all reagents from Gibco, Waltham, MA, USA). Cultures were maintained in a humidified atmosphere with 5% CO_2_ at 37 °C in a CO_2_ incubator (Thermo Fisher Scientific, Waltham, MA, USA).

#### 4.3.2. Differentiation of Monocytes into Macrophage-like Cells

To differentiate THP-1 monocytes into macrophage-like cells, suspension cultures (1 × 10^6^ cells/mL) were seeded into 6-well plates and treated with 100 ng/mL PMA (Sigma-Aldrich, St. Louis, MO, USA) for 96 h. Following this period, the PMA-containing medium was removed, and the cells were washed twice with phosphate-buffered saline (PBS) before being placed in a fresh medium free of PMA for 24 h to allow for recovery and stabilisation of the macrophage-like phenotype.

#### 4.3.3. Induction of Inflammation and Treatment with the Investigated Substance

After the recovery period, the cells were treated with lipopolysaccharide (LPS from E. coli O111:B4, Sigma-Aldrich, USA) at a concentration of 100 ng/mL to induce an inflammatory response. Simultaneously, HTLP was added to the culture medium at concentrations of 10, 50, and 100 µg/mL (the concentrations were chosen based on preliminary cytotoxicity studies demonstrating the absence of toxic effects at these doses). Control cultures were treated with PBS (negative control) or with LPS alone (positive inflammation control). The cells were incubated in the presence of LPS and HTLP for 24 h in three biological replicates for each condition, after which samples were collected for subsequent gene expression analysis.

#### 4.3.4. Gene Expression Analysis by Real-Time Quantitative PCR

##### Primer Design

Specific primers for real-time quantitative PCR were designed using the online tool NCBI Primer-BLAST. The design parameters included an oligonucleotide length of 18–25 bases, a melting temperature of 58–62 °C, a GC content of 40–60%, and an amplicon length of 80–150 base pairs. Primer specificity was confirmed using the BLAST (https://blast.ncbi.nlm.nih.gov/Blast.cgi (accessed on 13 August 2025)) algorithm to exclude the possibility of nonspecific amplification.

For the study, genes related to inflammatory processes (*TNF*, *IL1B*, *IL6*, *IL10*, *IL12B*, *IL23*, *CD40*, *CD80*, *CD274*) and cell development, survival, and longevity (*CSF-1*, *NAMPT*) were selected to evaluate the impact of HTLP on various aspects of the cellular response during inflammation. The sequences of the primers used are provided in Table 1.

##### RNA Extraction and Reverse Transcription

Total RNA was extracted from the samples using the phenol–chloroform extraction method with a commercial reagent kit (Evrogen, Moscow, Russia) according to the manufacturer’s instructions. The quality of the extracted RNA was evaluated spectrophotometrically at wavelengths of 260 and 280 nm using a NanoDrop (Thermo Fisher Scientific, Waltham, MA, USA); an A260/A280 ratio > 1.8 was considered indicative of high purity. Additionally, RNA integrity was confirmed by electrophoresis in a 1% agarose gel.

Complementary DNA (cDNA) synthesis was performed using the MMLV reverse transcriptase (Evrogen, Moscow, Russia). The reaction mixture (total volume 20 µL) contained 1–2 µg total RNA, oligo(dT) primers (20 µM), a mixture of deoxynucleotide triphosphates (10 mM), dithiothreitol (20 mM), and 100 units of reverse transcriptase. The reverse transcription reaction was carried out at 40 °C for 45 min, followed by enzyme inactivation at 70 °C for 10 min.

##### Real-Time Quantitative PCR

The obtained cDNA samples served as templates for real-time quantitative PCR. The reaction mixture consisted of 3 µL of cDNA (diluted 1:10), specific primers (10 µM each), and a commercial mix with SYBR Green and thermostable DNA polymerase (Evrogen, Moscow, Russia). Amplification was carried out on a DTlite device (DNA-Technology, Moscow, Russia) with the following programme: initial denaturation at 95 °C for 5 min, followed by 40 cycles of denaturation (95 °C for 30 s), primer annealing (58–62 °C for 30 s, depending on the primers), and elongation (72 °C for 30 s). Each sample was tested in three technical replicates.

Melting curve analysis was performed following amplification (temperature range 65–95 °C with a 0.5 °C increment) to confirm the specificity of the PCR products.

##### Data Analysis and Quality Control

To rule out genomic DNA contamination, each experiment had a negative control without reverse transcriptase (RT-) and a PCR negative control without a template. The absence of a signal in these samples demonstrated the purity of the RNA preparations and reaction mixtures.

Gene expression analysis was carried out on samples from four independent animals per group. Three technical replicates (qPCR wells) for each gene were analysed per biological sample. The non-parametric Wilcoxon signed-rank test (with Bonferroni correction) was used to compare gene expression levels in the LPS-only group versus each HTLP treatment group. *p*-values < 0.05 indicated statistical significance.

The target genes’ relative expression levels were determined using the 2^ΔΔCt^ method. GAPDH, which encodes glyceraldehyde-3-phosphate dehydrogenase, was used as a reference gene for normalisation; its expression stability was confirmed under experimental conditions [52,53]. The PCR efficiency for each gene was calculated using serial dilutions of cDNA and ranged between 95% and 105%.

### 4.4. Statistical Analysis

RStudio (2025.05.1 Build 513) software was used to conduct statistical analyses. The normality of the data distribution was assessed using the Shapiro–Wilk test. To quantify the rate of development of the anti-inflammatory effect, we calculated the percentage of inflammation inhibition (*PI*) and degree of inflammation (*DI*) over time. One-way ANOVA followed by Tukey’s post hoc test was used to compare differences between groups at each time point. A *p*-value < 0.05 was considered statistically significant. All values are expressed as mean ± SEM (*n* = 8 per group). Kruskal–Wallis and Mann–Whitney tests were used to compare oedema in different groups.

## 5. Conclusions

The presented data show that the polysaccharide HTLP has a potent and multifaceted anti-inflammatory effect. HTLP effectively reduced oedema in several inflammatory models, including carrageenan- and formalin-induced oedema and the pocket granuloma model, with efficacy approaching that of ibuprofen. In vivo activity results are consistent with molecular data, confirming that HTLP modulates the inflammatory cascade by suppressing pro-inflammatory mediator production while activating cellular defence mechanisms, as evidenced by increased anti-inflammatory factor expression.

Further research into HTLP and its molecular and cellular mechanisms of biological activity may lead to the development of new drugs for the treatment of inflammatory diseases in the future.

## Figures and Tables

**Table 1:** Primer sequences for real-time quantitative PCR.

Gene (Protein)	Forward Primer (5′→3′)	Reverse Primer (5′→3′)
*IL1B (IL-1β)*	ATGATGGCTTATTACAGTGGCAA	GTCGGAGATTCGTAGCTGGA
*IL6*	ACTCACCTCTTCAGAACGAATTG	CCATCTTTGGAAGGTTCAGGTTG
*IL10*	GACTTTAAGGGTTACCTGGGTTG	TCACATGCGCCTTGATGTCTG
*IL12B*	GCGGAGCTGCTACACTCTC	CCATGACCTCAATGGGCAGAC
*IL23*	CTCAGGGACAACAGTCAGTTC	ACAGGGCTATCAGGGAGCA
*TNFA (TNF-α)*	CCTCTCTCTAATCAGCCCTCTG	GAGGACCTGGGAGTAGATGAG
*CD40*	TTGGGGTCAAGCAGATTGCTA	GCAGATGACACATTGGAGAAGA
*CD80*	GGCACATACGAGTGTGTTGT	TCAGCTTTGACTGATAACGTCAC
*CD274*	GGACAAGCAGTGACCATCAAG	CCCAGAATTACCAAGTGAGTCCT
*CSF1*	TGGCGAGCAGGAGTATCAC	AGGTCTCCATCTGACTGTCAAT
*NAMPT*	GGCACCACTAATCATCAGACCTG	AAGGTGGCAGCAACTTGTAGCC

## Data Availability

The data presented in this study are available on request from the corresponding author.

## Abbreviations

The following abbreviations are used in this manuscript:

HTLP	*Helianthus tuberosus* L. polysaccharide
CSF	Colony-stimulating factor
cDNA	Complementary DNA
IFN	Interferon
IL	Interleukin
TNF	Tumour necrosis factor
IQR	Interquartile range
